# Functional Analysis of the Cathepsin D Gene Response to SGIV Infection in the Orange-Spotted Grouper, *Epinephelus coioides*

**DOI:** 10.3390/v14081680

**Published:** 2022-07-29

**Authors:** Yuexuan Wang, Honglin Han, Kecheng Zhu, Suifeng Xu, Chengzong Han, Yunxiang Jiang, Shina Wei, Qiwei Qin

**Affiliations:** 1 Laboratory for Lingnan Modern Agriculture, College of Marine Sciences, South China Agricultural University, Guangzhou 510642, China; wangyuexuan@stu.scau.edu.cn (Y.W.); hanhonglin@stu.scau.edu.cn (H.H.); xusuifeng@stu.scau.edu.cn (S.X.); czhan@stu.scau.edu.cn (C.H.); jiangyunxiang@stu.scau.edu.cn (Y.J.); 2 Key Laboratory of South China Sea Fishery Resources Exploitation and Utilization, Ministry of Agriculture and Rural Affairs, South China Sea Fisheries Research Institute, Chinese Academy of Fishery Sciences, Guangzhou 510300, China; zkc537@163.com; 3 Southern Marine Science and Engineering Guangdong Laboratory (Zhuhai), Zhuhai 528478, China; 4 Laboratory for Marine Biology and Biotechnology, Qingdao National Laboratory for Marine Science and Technology, Qingdao 266000, China

**Keywords:** Singapore grouper iridovirus (SGIV), grouper, cathepsin D, apoptosis

## Abstract

(1) Background: Lysosomal aspartic protease Cathepsin D (CD) is a key regulator and signaling molecule in various biological processes including activation and degradation of intracellular proteins, the antigen process and programmed cell death. However, the function of fish CD in virus infection remains largely unknown. (2) Methods: The functions of the CD gene response to SGIV infection was determined with light microscopy, reverse transcription quantitative PCR, Western blot and flow cytometry. (3) Results: In this study, Ec-Cathepsin D (Ec-CD) was cloned and identified from the orange-spotted grouper, *Epinephelus coioides*. The open reading frame (ORF) of Ec-CD consisted of 1191 nucleotides encoding a 396 amino acid protein with a predicted molecular mass of 43.17 kDa. Ec-CD possessed typical CD structural features including an N-terminal signal peptide, a propeptide region and a mature domain including two glycosylation sites and two active sites, which were conserved in other CD sequences. Ec-CD was predominantly expressed in the spleen and kidneys of healthy groupers. A subcellular localization assay indicated that Ec-CD was mainly distributed in the cytoplasm. Ec-CD expression was suppressed by SGIV stimulation and Ec-CD-overexpressing inhibited SGIV replication, SGIV-induced apoptosis, caspase 3/8/9 activity and the activation of reporter gene p53 and activating protein-1 (AP-1) in vitro. Simultaneously, Ec-CD overexpression obviously restrained the activated mitogen-activated protein kinase (MAPK) pathways, including extracellular signal-regulated kinase (ERK) and c-Jun N-terminal kinase (JNK). In addition, Ec-CD overexpression negatively regulated the transcription level of pro-inflammatory cytokines and activation of the NF-κB promotor. (4) Conclusions: Our findings revealed that the Ec-CD possibly served a function during SGIV infection.

## 1. Introduction

Cathepsins are widely presented in eukaryotic cells and are roughly composed of cysteine protease (cathepsin B, C, F, H, K, L, O, S, W, and Z), aspartic protease (cathepsin D and E) and serine protease (cathepsin A and G) [1]. Most Cathespins are localized in lysosomal acidic vesicles and depend on the acidic pH environment to exert their catalytic activity. Cathepsin D (CD), which accounts for 11% of proteases in the lysosome, has been characterized as a pepsin of the aspartic proteinase family and for its function in shearing and degrading peptide chains [2,3,4], participating in antigen presentation and regulating cell cycle progression [5,6,7]. Moreover, much research has investigated the role of CD in disease. For instance, CD can maintain neuronal cell homeostasis via mediating proteolysis [8]. In human disease, CD was deemed to be a remedy related to candidate targets in breast carcinoma and Alzheimer’s disease [9,10]. Given its regulatory role in physiology, CD inhibitors such as Pepstatin A [11] and Tasiamide B [12,13], and synthetic derivatives of Tasiamide B [14,15,16], have been developed. So far, CD has been identified in bony fish such as lampreys, grass carp and rock breams [17,18,19]. However, the limitation of current research is that it typically focuses on the role of CD in mammalian physiological activities. Studies rarely focus on the features of CD in the antiviral response to DNA virus invasion.

The orange-spotted grouper (*Epinephelus coioides*), belonging to the *Perciformes*, *Serranidae*, is favored by consumers and farmers because of its delicious meat and rich nutrition, and is one of the main farmed fish in China. Singapore grouper iridovirus (SGIV), a high fatality rate virus disease, resulted in enormous economic loss to the aquaculture industry [17,18,19,20,21]. We previously found that cathepsins were the key element in the fight against SGIV infection; however, whether CD is involved in antiviral response is unclear [21,22,23].

In this study, after cloning and characterizing Ec-Cathepsin D (Ec-CD), we analyzed its expression pattern, subcellular localization, roles in viral infection and the impact on cellular innate immunity. Taken collectively, these findings revealed the crucial role of grouper CD in antiviral innate immunity.

## 2. Materials and Methods

### 2.1. Fish, Cells and Virus

Grouper spleen (GS) cells were cultured in Leibovitz’s L15 medium supplemented with 10% fetal bovine serum (Gibco, Waltham, MA, USA) at 28 °C, as well as fathead minnow (FHM) epithelial cells [24,25]. SGIV was prepared as described previously [21].

### 2.2. Cloning of Ec-CD and Bioinformatic Analysis

The primers Ec-CD-F/Ec-CD-R (Table 1) were used in polymerase chain reaction (PCR) condition for obtaining the open reading frame (ORF) of Ec-CD, which was based on the expressed sequence tag (EST) sequences of grouper CD. The Conserved Domains program (https://www.ncbi.nlm.nih.gov/cdd/, accessed on 10 May 2022) and the BLAST program (http://www.ncbi.nlm.nih.gov/blast, accessed on 10 May 2022) were used to analyze the sequence and predicted the specific construction of Ec-CD, respectively. Clustal X1.83 software, the GeneDoc program and the MEGA 6.0 software were used for the amino acid alignments and phylogenetic analysis.

### 2.3. Virus Infection Assay

We infected GS cells and FHM cells with SGIV (multiplicity of infection (MOI) = 0.1) to elucidate the changes of Ec-CD during SGIV stimulation. A light microscope (Zeiss, Oberkochen, Germany) was used to visualize viral cytopathic effects and cells were harvested for subsequent experiments.

### 2.4. Ec-CD Expression Patterns

To probe the distribution of Ec-CD in tissues, spleen, muscle, head kidney, liver, brain, gill, heart and kidney were gathered from fit groupers for qRT-PCR analysis. To explore the influence of SGIV invasion on Ec-CD, GS cells were gathered at SGIV post-infection for subsequent experiments.

### 2.5. Plasmid Construction and Cell Transfection

ORFs of Ec-CD sub-cloned into pEGFP-C1 vectors (Invitrogen, Los Angeles, CA, USA) to generate recombinant plasmids pEGFP-CD. Primers used are listed in Table 1. Based on the previous method [26], Lipofectamine 2000 transfection reagent (Invitrogen, Los Angeles, CA, USA) was used in cell transfection. Briefly, monolayer cells were incubated with Lipofectamine 2000 and plasmids in serum-free medium. At 6 h post-incubation, serum-free medium was changed to serum-containing medium.

### 2.6. Cellular Localization Assay

Four percent paraformaldehyde and 4,6-diamidino-2-phenylindole (DAPI) were manipulated to fix and stain transfected GS cells, respectively. Fluorescence signals were imaged under fluorescence microscopy (Leica, Wetzlar, Germany).

### 2.7. Ec-CD Knockdown Analysis

To explore the effects of Ec-CD knockdown on virus infection in cells, Small Interfering RNA (siRNA) targeting three sequences of Ec-CD mRNA was synthesized by GenePharma, Shanghai, China (Table 1). In addition, cells were transfected with siRNAs, and Si-Cathepsin D-341 was used as si-Cathepsin D in follow-up experiments.

### 2.8. RNA Extraction and qRT-PCR

A Cell Total RNA Isolation Kit (FORE GENE, Chengdu, China) was used to extract total RNA, and a ReverTra Ace qPCR RT Kit (TOYOBO, Osaka, Japan) was used for the reverse transcription of RNA, as described previously [26]. An Applied Biosystems QuantStudio 5 Real Time Detection System (Thermo Fisher, Waltham, MA, USA) with the cyclic conditions described previously was used for qRT-PCR analysis [22]. The primers used in the experiment are listed in Table 2. The relative expression ratio of the selected gene vs. β-actin (reference gene) was calculated using the 2^−∆∆Ct^ method.

### 2.9. Reporter Gene Assay

GS cells in each of the 48-well plates were co-transfected with 300 ng pEGFP-C1 or pEGFP-CD and reporter gene plasmids containing 150 ng NF-κB-Luc and 15 ng SV40. In the apoptosis assay, FHM cells were co-transfected with 300 ng pEGFP-CD or pEGFP-C1 and luciferase plasmids including 15 ng SV40 and 150 ng AP1-Luc or P53-Luc. Luciferase activities in cells were detected by the Dual-Luciferase^®^ Reporter Assay System (Promega, Madison, WI, USA).

### 2.10. Western Blot Analysis

To detect the synthesis of relative protein, GS cells were lysed with Pierce IP Lysis Buffer (Beyotime, Shanghai, China). Next, 10% SDS-PAGE was used to isolate proteins and Immobilon-polyvinylidene difluoride membranes (Millipore, Temecula, CA, USA) with transferred proteins, which were incubated in 5% bovine serum albumin (BSA) and then in different antibodies. Specific primary antibodies against β-tubulin (1:2000 dilution, Abcam, Cambridge, UK) served as a loading control, SGIV major capsid protein (MCP) (1:4000 dilution), SGIV ORF162 (1:2000 dilution), ERK1/2 (1:1000 dilution, CST), JNK (1:1000 dilution, CST), phospho-ERK1/2 (1:1000 dilution, CST), phospho-SAPK/JNK (1:1000 dilution, CST) and cleaved caspase3 (1:1000 dilution, CST) in the experiment. After being washed in TBST for 15 min, peroxidase-conjugated affinipure goat anti-rabbit IgG (1:6000 dilution, Abcam) was tested, and immune-reactive proteins were visualized by enhanced chemiluminescence (Thermo Fisher, Waltham, MA, USA).

### 2.11. Cell Apoptosis Analysis

To demonstrate the effect of Ec-CD on SGIV-induced cell apoptosis, the percentage of apoptotic cells was detected by flow cytometry analysis according to the previous method [27]. In brief, FHM cells transfected with pEGFP-C1/CD or si-Cathepsin D were treated with SGIV infection and then gathered and fixed in 70% ethanol. A buffer solution containing propidium iodide (PI; Sigma-Aldrich, St. Louis, MO, USA) was utilized to stain centrifuged cells. FlowJo software was used to analyze the data obtained by flow cytometry. A Caspase Glo^®^ 3/8/9 Assay kit (Promega, Madison, WI, USA) was used to detect the changes of caspase 3/8/9 activation. FHM cells transfected with pEGFP-C1 or pEGFP-CD were infected with SGIV for 12 h or 24 h and then equal volumes as the culture medium of the reagent were added to samples for reactions. At 1 h post-infection, a microplate reader (Thermo Fisher, Waltham, MA, USA) was used to measure levels of caspase activation.

### 2.12. Statistical Analysis

GraphPad Prism 9 software was used to perform all the data analysis, and date were expressed as means ± the standard deviations (SD). Student’s *t*-test was used for statistical comparisons, and differences between the means were considered significant at *p* < 0.05.

## 3. Results

### 3.1. Sequence Characterization of Ec-CD

The ORF of Ec-CD consisted of 1191 nucleotides that encoded a 396 amino acid protein. Ec-CD included one N-terminal signal peptide (1 aa−18 aa) and one propeptide (19 aa−61 aa). Two key active sites were contained in the amino acid sequence of Ec-CD at position 94 aa and 281 aa, respectively. Moreover, two glycosylation sites were localized at position 131 aa and 249 aa, respectively (Figure S1A). Ec-CD shared 99.5% identity with the CD homolog of the giant grouper (*Epinephelus lanceolatus*), but 62.9% compared to humans (*Homo sapiens*). Phylogenetic analysis implied that Ec-CD showed the closest relationship to that of *Epinephelus*, and all the fish Ec-CD were clustered into one group separated from amphibians, birds and mammals (Figure S1B).

### 3.2. Tissue Distribution, Expression Profiles and Subcellular Distribution of Ec-CD

**Figure 1:**
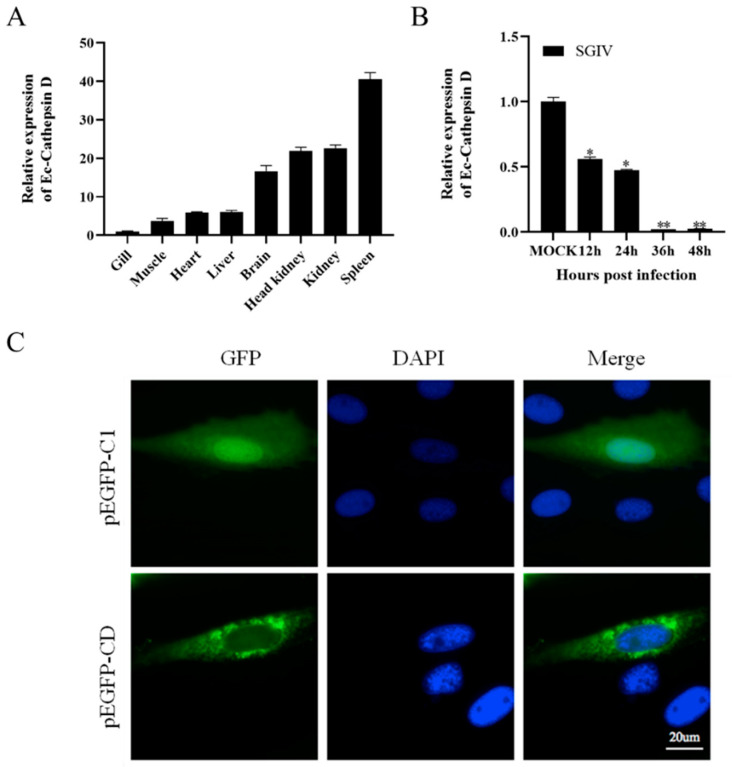
Expression patterns of Ec-CD were determined by qRT-PCR analysis. (**A**) The expression level of Ec-CD in different tissues from healthy groupers. Data are expressed as a ratio to the Ec-CD mRNA expression in gills. (**B**) GS cells were infected with SGIV and the expression level of Ec-CD was analyzed at different time points. β-actin (reference gene) was calculated using the 2^−^^∆∆Ct^ method. Results are presented as the means ± SD of data from four independent experiments. * *p* < 0.05, ** *p* < 0.01. (**C**) Subcellular localization of Ec-CD in grouper cells. PEGFP-C1 and pEGFP-CD were transfected into GS cells. After 24 h, cells were fixed and stained with DAPI and imaged by fluorescence microscopy. Green fluorescence indicated pEGFP-C1 or pEGFP-CD, and blue fluorescence indicated the nucleus.

The relative expression of Ec-CD in different tissues from healthy juvenile, orange-spotted groupers was analyzed by qRT-PCR. The results revealed that Ec-CD was distributed in eight detection tissues and highly expressed in the spleen, kidney, head kidney and brain (Figure 1A). In response to SGIV simulation, the mRNA expression of Ec-CD was obviously down-regulated (Figure 1B). To evaluate the role of Ec-CD, subcellular location was tested in GS cells. PEGFP-C1 and the recombinant plasmid, pEGFP-CD, were transfected into GS cells, respectively. As shown in Figure 1C, the green fluorescent signals representing pEGFP-C1 was equally distributed in the whole cell, whereas in pEGFP-CD-transfected cells, the fluorescence signal was mainly observed in the cytoplasm, while some was observed with dot-like aggregation forms near the nucleus, which might imply that the function of Ec-CD was associated with vesicles (Figure 1C).

### 3.3. Overexpression of Ec-CD Inhibited SGIV Infection

**Figure 2:**
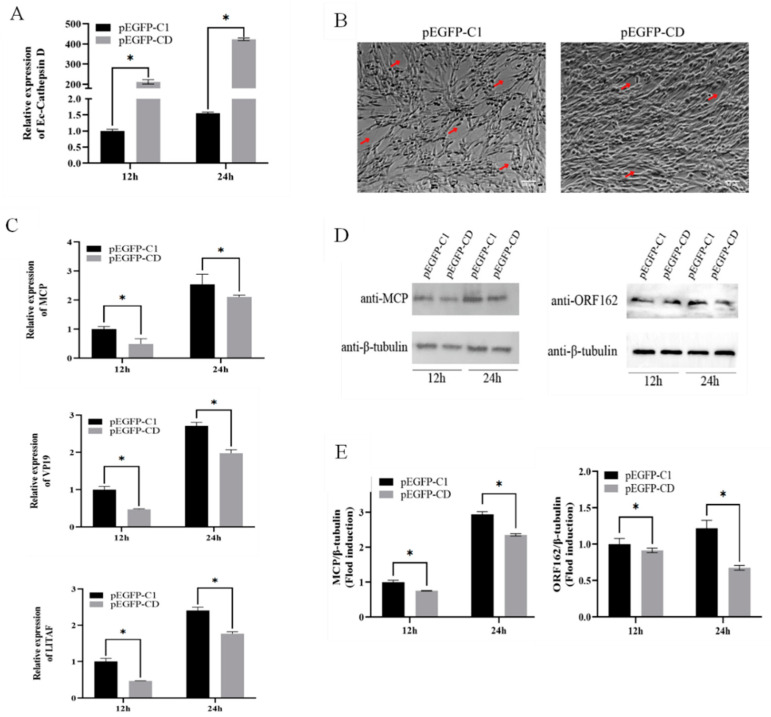
Overexpression of Ec-CD significantly inhibited SGIV infection. Cells were infected with SGIV after being transfected with pEGFP-C1 and pEGFP-CD 24 h later. (**A**) The relative expression of Ec-CD was evaluated by qRT-PCR. (**B**) The severity of CPE induced by SGIV was weakened. (**C**) The relative expressions of SGIV-MCP, SGIV-VP19 and SGIV-LITAF genes after SGIV infection were evaluated by qRT-PCR. The relative expression ratio of the selected gene vs. β-actin (reference gene) was calculated using the 2^-^^∆∆Ct^ method. (**D**) SGIV-MCP protein SGIV-ORF162 protein and cellular β-tubulin were analyzed by WB, and MCP/β-tubulin, ORF162/β-tubulin for each group were calculated as above. (**E**). Data are means ± SD from four experiments. Student’s *t*-test: * *p* < 0.05.

To elucidate the impact of Ec-CD on SGIV invasion, GS cells transfected pEGFP-C1 or pEGFP-CD were infected with SGIV and then the viral gene replication was investigated. The relative expression of Ec-CD was detected to prove the success of overexpression. QRT-PCR showed that the expression of Ec-CD in the cells transfected with pEGFP-CD was 200 times and 270 times higher, than that in the control group at 12 h or 24 h, respectively (Figure 2A). In addition, the expression changes of viral capsid proteins-MCP, envelope proteins-VP19 and early genes-LITAF and ORF162 were detected. Results suggested that Ec-CD overexpression significantly weakened SGIV-related CPE, and the mRNA levels of SGIV-MCP, SGIV-VP19 and SGIV-LITAF decreased (Figure 2B). Western blot indicated that SGIV-MCP and SGIV-ORF162 protein synthesis was decreased in cells which overexpressed Ec-CD (Figure 2C,D). In summary, the overexpression of Ec-CD inhibited the SGIV infection.

### 3.4. Overexpression of Ec-CD Hindered SGIV-Induced Cell Apoptosis

**Figure 3:**
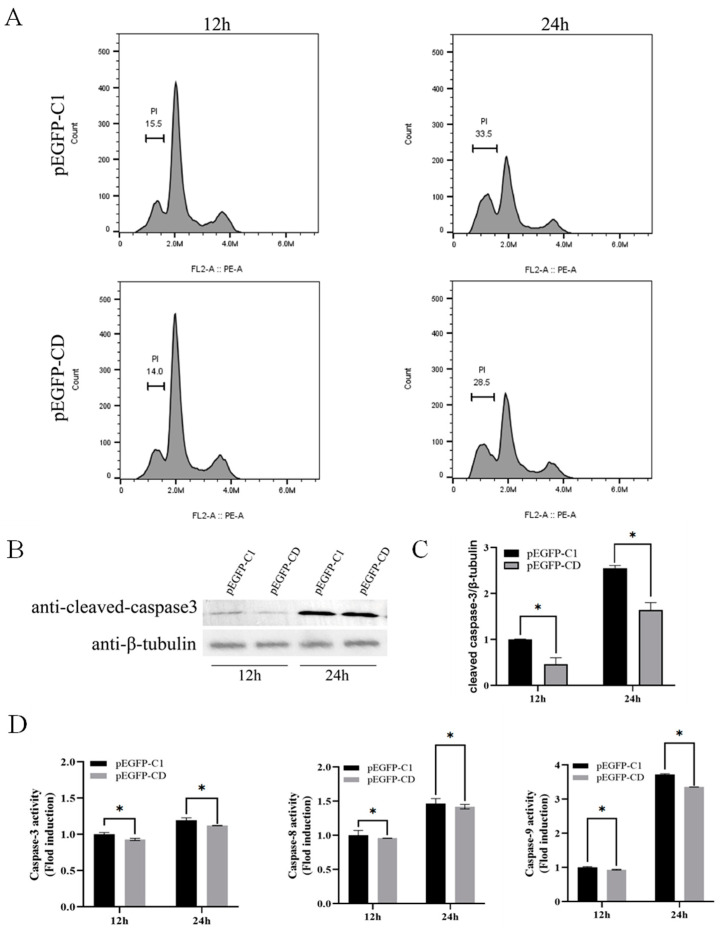

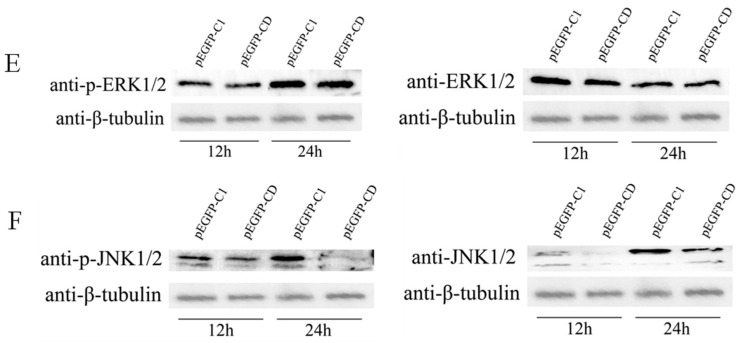
The effect of Ec-CD overexpression on apoptosis of fathead minnow (FHM) cells during SGIV infection. (**A**) Flow cytometry analysis of DNA content in SGIV-infected FHM cells. The percentage of the sub-G0/G1 phase was calculated and is indicated on the histogram. (**B**) The level of cleaved caspase-3 proteins was detected by WB, and cleaved caspase-3/β-tubulin for each group was calculated as above (**C**). (**D**) The activities of caspase-3, caspase-8 and caspase-9 were examined in SGIV-infected FHM. Date are expressed as a ratio to the caspase activity of cells transfected with pEGFP-C1 at 12 h. Data are means ± SD from four experiments. Student’s *t*-test: * *p* < 0.05. (**E**) Detection of ERK1/2 phosphorylation and (**F**) JNK1/2 phosphorylation expression during SGIV infection by WB. Cell lysates from SGIV-infected FHM cells transfected with pEGFP-C1 or pEGFP-CD were collected at the indicated times and subjected to WB. To verify equal loading, Western blotting was performed with antibody against β-tubulin, ERK1/2, JNK, phospho-ERK1/2, and phospho-SAPK/JNK.

**Figure 4:**
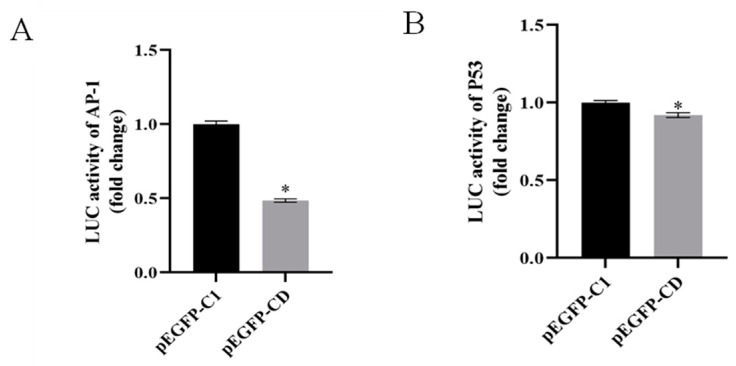
Ec-CD overexpression inhibited the luciferase activities of activating protein-1 (AP-1) and p53 promoters. (**A**) FHM cells were co-transfected with AP-1-Luc and pEGFP-C1 or pEGFP-CD, and then cells were infected with SGIV at 24 h post-transfection. The luciferase reporter gene assay was used to detect the activities of AP-1 promoters. (**B**) FHM cells were co-transfected with P53-Luc and pEGFP-C1 or pEGFP-CD, and then cells were infected with SGIV at 24 h post-transfection. The luciferase reporter gene assay was used to detect the activities of P53 promoters. Data are means ± SD from four experiments. Student’s *t*-test: * *p* < 0.05.

To access the effects of Ec-CD overexpression on cell apoptosis, we selected FHM cells as a research model in which cell apoptosis occurred when cells were infected with SGIV [28]. Results of flow cytometry showed that SGIV-induced a sub-G0/G1 peak, indicating the apoptosis of cells at 24 h was higher than at 12 h; Ec-CD expression decreased the peak of apoptosis at both 12 h and 24 h compared with the control group (Figure 3A). Western blot results indicated that protein synthesis of cleaved caspase-3 was reduced in cells overexpressing Ec-CD (Figure 3B,C). Compared with the control group, the activity of caspase-3, caspase-8 and caspase-9 were all lower (Figure 3D). To further explore the molecular mechanism of inhibiting apoptosis by Ec-CD overexpression, the ERK and SAPK/JNK signaling pathway related to virus-induced cell death was detected by Western blots. The synthesis of total ERK1/2 protein and the phosphorylation of ERK1/2 were both significantly decreased in cells transfected with pEGFP-CD (Figure 3E). Similarly, total protein and phosphorylated proteins of SAPK/JNK were reduced in cells overexpressing Ec-CD (Figure 3F). The effect of Ec-CD on critical transcription factors related to cell apoptosis, including AP-1 and P53, were detected using the luciferase reporter gene assay. The results revealed that Ec-CD overexpression resulted in adverse effects on the activations of AP-1 (Figure 4A) and P53 (Figure 4B) at 24 h. The fold changes were reduced by 51.5 and 9.8%, respectively.

### 3.5. Ec-CD Knockdown Promoted SGIV Infection

**Figure 5:**
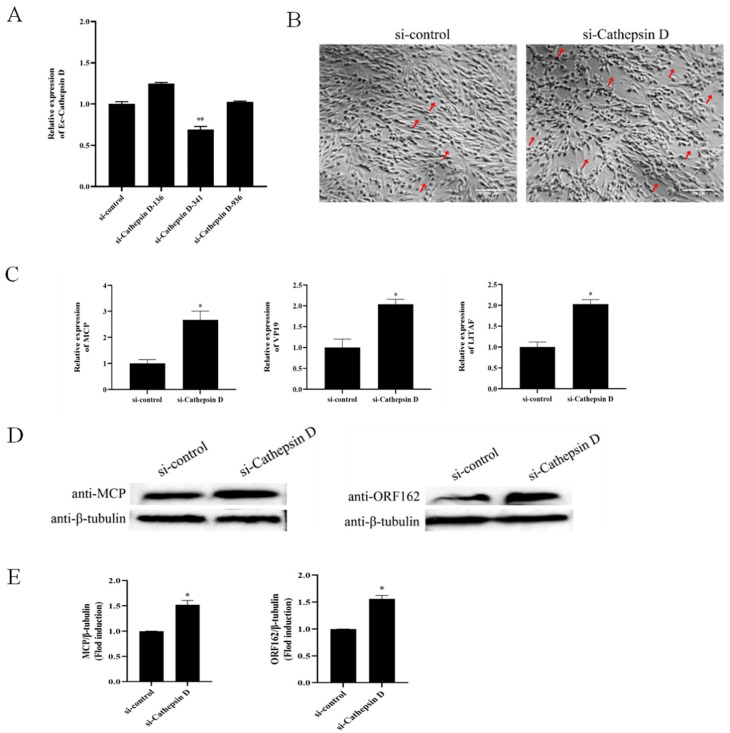
Knockdown of Ec-CD promoted SGIV infection. (**A**) The knockdown efficiency of si-Cathepsin D. After GS cells were transfected with si-control or si-Cathepsin D for 24 h, cells were harvested for qRT-PCR. (**B**) GS cells were infected SGIV after being transfected with si-control or si-Cathepsin D. Knockdown of Ec-CD reduced the CPE induced by SGIV. The red arrows show CPE. (**C**) The relative expressions of SGIV-MCP, SGIV-VP19 and SGIV-LITAF genes after SGIV infection were evaluated by qPCR. The relative expression ratio of the selected gene vs. β-actin (reference gene) was calculated using the 2^-^^∆∆Ct^ method. (**D**) Detection of SGIV-MCP and SGIV-ORF162 expression during SGIV infection by WB. and MCP/β-tubulin, ORF162/β-tubulin for each group were calculated as above (**E**). Data are means ± SD from four experiments. Student’s *t*-test: * *p* < 0.05.

Given the role of Ec-CD overexpression on SGIV invasion, we explored the influence of Ec-CD by si-RNA interference to further confirm the results. The efficient knockdown of Ec-CD was detected by qRT-PCR, which showed that at 24 h the expression of Ec-CD in GS cells transiently transfected with si-Cathepsin D-341 was reduced (Figure 5A). Hence, si-Cathepsin D-341 is regarded as si-Cathepsin D. Cells transfected with si-Cathepsin D were detected after being infected with SGIV for 24 h. Results suggest that the CPE caused by SGIV was aggravated (Figure 5B), and the mRNA expression of SGIV-MCP, SGIV-VP19 and SGIV-LITAF increased (Figure 5C) in si-Cathepsin D- transfected cells. Western blot showed that SGIV-MCP and SGIV-ORF162 protein synthesis was promoted in si-Cathepsin D- transfected cells (Figure 5D,E). These data indicated that Ec-CD knockdown promoted SGIV infection.

### 3.6. Ec-CD Knockdown Promoted SGIV-induced Cell Apoptosis

**Figure 6:**
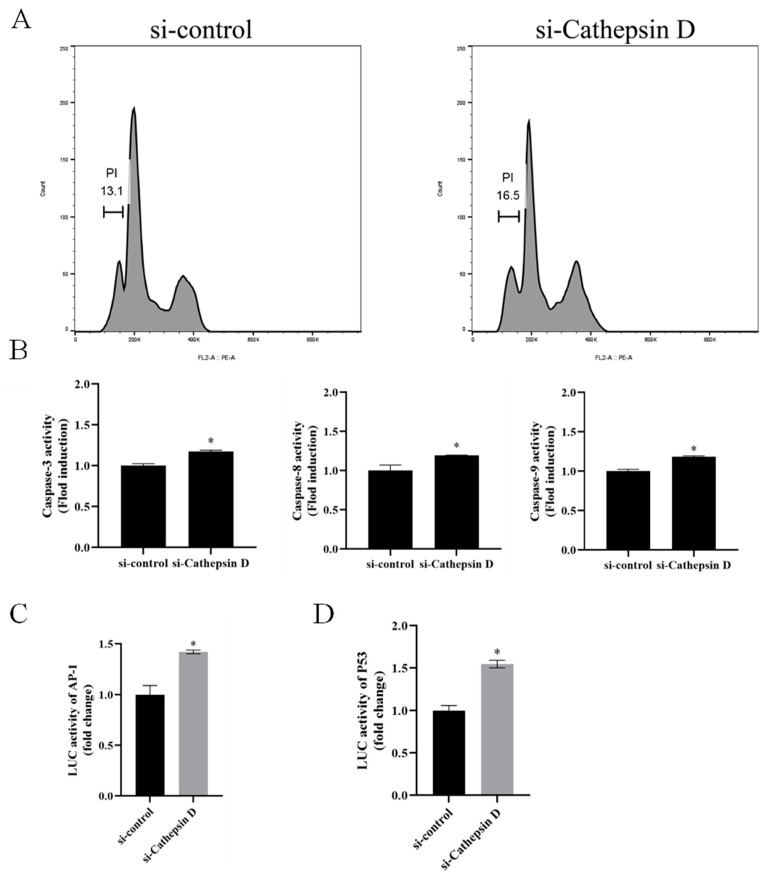
Knockdown of Ec-CD promoted apoptosis. FHM cells were infected with SGIV after being transfected with si-control or si-Cathepsin D. (**A**) The percentage of apoptotic cells in the hypodiploid DNA peak (sub-G1 population) was calculated by sub-G1 population/total cell cycle populations. (**B**) The activity of caspase-3, caspase-8 and caspase-9 after SGIV infection. (**C**,**D**) FHM cells were co-transfected with AP-1-Luc or P53-Luc and si-Cathepsin D, and then cells were infected with SGIV at 24 h post-transfection. The luciferase reporter gene assay was used to detect the activities of the AP-1 or P53 promoters. Control group: cells were co-transfected with AP-1-Luc or P53-Luc and si-control. Data are means ± SD from four experiments. Student’s *t*-test: * *p* < 0.05.

To explore whether Ec-CD knockdown impacted SGIV-induced cell apoptosis, Si-Cathepsin D was transfected into FHM cells and the cells were collected after being infected with SGIV for 24 h for cell apoptosis analysis, caspase-3/8/9 activity analysis and dual luciferase reporter assays. Flow cytometry analysis showed that the percentage of apoptosis cells increased in si-Cathepsin D transfected cells (Figure 6A). The activities of caspase-3, caspase-8 and caspase-9 were all higher than that in the control group (Figure 6B). Ec-CD knockdown promoted the activations of AP-1 (Figure 6C) and P53 (Figure 6D). These results demonstrate that Ec-CD knockdown promoted SGIV-induced cell apoptosis.

### 3.7. Ec-CD Knockdown Promoted SGIV-induced Cell Apoptosis

**Figure 7:**
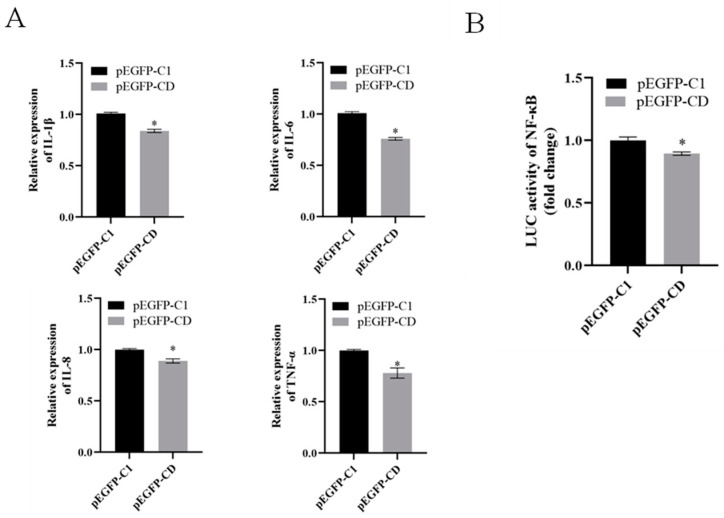
Ec-CD overexpression inhibited the pro-inflammatory signaling molecules. (**A**) GS cells were transfected with pEGFP-C1 or pEGFP-CD for 24 h, followed by the transcription of IL-1β, IL-6, IL-8 and TNF-α, which were analyzed by qRT-PCR. (**B**) GS cells were co-transfected with NF-κB-Luc and pEGFP-C1 or pEGFP-CD for 24 h, and then the activities of NF-κB promoters were detected. Data are means ± SD from four experiments. Student’s *t*-test: * *p* < 0.05.

To verify the roles of Ec-CD in inflammatory responses, we examined the expression of proinflammatory cytokines including IL-1β, IL-6, IL-8 and TNF-α by qRT-PCR and the promoter activities of NF-κB related to inflammatory genes expression in GS cells transfected with Ec-CD-overexpressing cells. The transcription levels of IL-1β, IL-6, IL-8 and TNF-α were all lower than that in Ec-CD overexpression cells (Figure 7A), and the overexpression of Ec-CD substantially decreased the activity of NF-κB promoters (Figure 7B). Based on the data above, we propose that Ec-CD impaired the inflammatory response.

## 4. Discussion

Cathepsin D (CD) is a highly conserved protease in the lysosomal system and relies on aspartic acid residues for its catalytic activity. Numerous studies have indicated that CD is involved in biological processes during pathogen invasion [3,29]. In this study, we cloned and characterized Ec-Cathepsin D (Ec-CD) and analyzed its expression in different tissues of grouper. Moreover, we analyzed the effect of SGIV infection on CD expression and investigated the effect of CD expression on SGIV replication and SGIV-induced apoptosis. Although the underlying mechanism remains to be explored, we revealed that Ec-CD had a regulatory function in SGIV infection.

Ec-CD was cloned and identified for the first time and was found to encode a 396 amino acid protein. Sequence analysis showed that it was highly conserved with other CD sequences in bony fish. Ec-CD shared the highest homology with giant grouper *Epinephelus lanceolatus* (99.5%), followed by Comb Rockfish *Sebastes umbrosus*, and shared 82.6% identity with zebrafish Danio rerio. In the phylogenetic tree, all teleost fish are grouped together, while birds and mammals are clustered into another group. Like CD sequences in other species, Ec-CD includes a signal peptide, a propeptide and a mature domain containing two glycosylation sites associated with proper transference and two critical catalytic activity sites [3,17,19]. In view of the above, it was speculated that Ec-CD plays a similar physiological function as in other species. However, studies have shown that in mammals, the mature CD contains a light chain and a heavy chain, which rely on hydrophobic interaction to form a β-sheet structure, while CD from fish and other non-mammalians has single-chain molecules due to the lack of amino acid residues necessary to generate a two-chain form [30,31]. To investigate the function of Ec-CD, we examined the subcellular localization of Ec-CD using a green fluorescent protein, and the results showed that green fluorescence representing Ec-CD was excluded from the nucleus, as is consistent with previous reports [5]. Cathepsins are a concern with the immune response to pathogen invasion [32]. To clarify the immune role of Ec-CD, we monitored the expression pattern of Ec-CD in different tissues of grouper and with virus stimulation. Ec-CD mRNA was abundantly expressed in the spleen of grouper, the main immune organ, followed by the kidney and the head kidney. Compared with uninfected virus, the transcription level of Ec-CD decreased significantly in GS cells (75% reduction at 48 h) as the SGIV infection time advanced. These changes suggest that Ec-CD might play a momentous role in the host’s antiviral innate immune response.

To further elucidate the molecular mechanism of Ec-CD involving the host immune response to viral infection, the effects of Ec-CD overexpression and knockdown on SGIV infection were explored. The results show that the transcription and protein synthesis of viral genes were impeded after Ec-CD overexpression, but were induced by Ec-CD knockdown. Viral infection could induce the secretion of proinflammatory factors by cells, which was confirmed to lead to complications and organism damage [29,33,34,35]. Accordingly, numerous reports have focused on the vital role of anti-inflammatory properties in defending against viral attack. For example, the application of Lianhuaqingwen in inhibiting SARS-CoV-2 infection was related to its anti-inflammatory activity [36], and anti-inflammatory factors are involved in the cGAS-STING defense pathway against viruses [37]. As in previous reports, Cathepsins could be a therapeutic target for inflammatory diseases [38], and the expression of pro-inflammatory cytokines in CD-overexpressing cells was examined to prove the impact of Ec-CD on the host immune response. The results reveal that transcript levels of pro-inflammatory cytokines were decreased in CD-overexpressing cells. Given that NF-κB can activate inflammatory responses [39,40,41], and it has been reported as a potential therapeutic target in metabolic disease and swine-origin influenza A (H1N1) virus [39,40,41,42], we subsequently carried out the fluorescein reporter gene assay and the results showed that Ec-CD reduced the promoter activity of NF-κB. These results suggest that Ec-CD can protect against SGIV damage by attenuating cellular inflammatory responses through the NF-κB pathway.

Apoptosis can serve as a host defense mechanism, while virus-induced apoptosis is associated with viral escape from host immune responses. Reports indicate that West Nile virus (WNV), and Japanese encephalitis virus (JEV) use apoptosis as a virulence factor to promote their pathogenesis [43,44,45]. The human immunodeficiency virus (HIV) envelope protein induced apoptosis in immune cells [46,47]. It is known that SGIV infection of FHM induces canonical apoptosis and facilitates the activation of caspase 3/8/9, which is associated with the activation of p53 and AP-1 pathways [48,49,50]. The involvement of CD in apoptosis has also been widely reported. CD can not only act on caspase8 to promote apoptosis, but also participate in the anti-apoptotic effect [51,52]. We overexpressed Ec-CD in FHM cells, analyzed Ec-CD influence on SGIV-induced apoptosis by flow cytometry and detected caspase activity. The results indicate that Ec-CD overexpression impaired apoptosis caused by SGIV and caspase-3/8/9 activity, while Ec-CD knockdown induced SGIV-induced apoptosis and stimulated caspase activity. At the same time, the Dual-luciferase reporter gene assay confirmed that the activities of the promoters AP-1 and p53 were both reduced in Ec-CD-overexpressing cells, while Ec-CD knockdown showed the opposite. Interestingly, it was also previously reported that in Tet21N cells, overexpression of CD attenuated doxorubicin-induced apoptosis [53]. Therefore, we speculated that Ec-CD could inhibit apoptosis by the AP-1 and p53 pathway.

The MAPK signaling pathway plays a key role in the process of cell death [54]. The ERK signal pathway was shown to be involved in SGIV- and soft-shelled turtle iridovirus (STIV)-induced apoptosis, and the JNK signal pathway was shown to be required for CIV-induced apoptosis [28,55,56]. To prove whether the inhibition of SGIV-induced apoptosis by Ec-CD involved the MAPK pathway, we examined the phosphorylation of ERK and JNK in Ec-CD-overexpressing cells. The results demonstrate that the overexpression of Ec-CD prevented the phosphorylation of ERK and JNK, suggesting that the ERK and JNK pathway responded to the regulation of Ec-CD on apoptosis. In addition, JNK1 can enhance the infectivity of SGIV and participate in blocking of the cellular immune response [50]. Consequently, the inhibition of JNK activation by Ec-CD is pivotal for resistance to SGIV infection.

## 5. Conclusions

**Figure 8:**
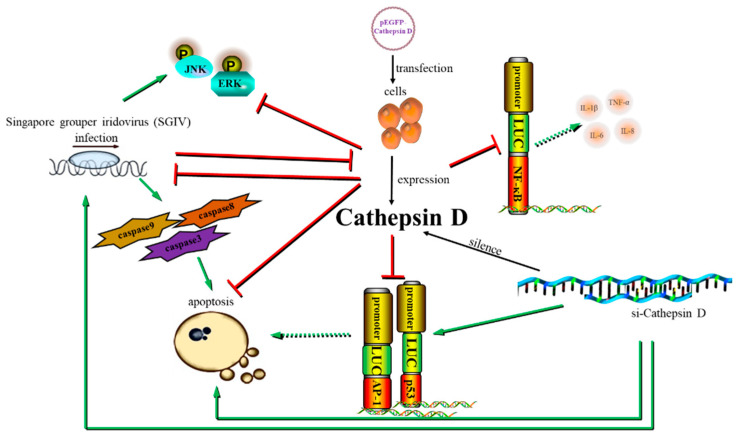
Schematic diagram of the regulation of Ec-CD during SGIV infection.

We cloned and characterized Ec-CD, and investigated the response of Ec-CD to SGIV invasion. As shown in Figure 8, Ec-CD expression was reduced by SGIV stimulation, and overexpression of Ec-CD inhibited SGIV infection and SGIV-induced apoptosis in vitro. In addition, Ec-CD negatively regulated the inflammatory responses. Our findings reveal that the regulation effects of the Ec-CD in host innate immune response during DNA virus invasion, and provides novel insights to the biological functions of Ec-CD.

## Figures and Tables

**Table 1:** Primers used for silencing Ec-Cathepsin D.

Primers	Sequences (5′–3′)
NC-F	UUCUUCGAACGUGUCACGUTT
NC-R	ACGUGACACGUUCGGAGAATT
Si-Cathepsin D-136-F	GCCGACACACACUCCCUUATT
Si-Cathepsin D-136-R	UAAGGGAGUGUGUGUCGGCTT
Si-Cathepsin D-341-F	GCUGGCUUCACCACAAAUATT
Si-Cathepsin D-341-R	UAUUUGUGGUGAAGCCAGCTT
Si-Cathepsin D-936-F	GGUGAACUGUGACAAGGUUTT
Si-Cathepsin D-936-R	AACCUUGUCACAGUUCACCTT

**Table 2:** Primers used for host and viral gene expression analysis.

Primers	Sequences (5′–3′)
C1-Ec-Cathepsin D-F	ATGAAGCTGTTGCTCCTCTTCGTGT
C1-Ec-Cathepsin D-R	TCACTTGGACTTGGCAAAGCCC
Ec-Cathepsin D-RT-F	GGTGCCCTCCGTTCACTGCTCCAT
Ec-Cathepsin D-RT-R	GCCCGACAAACTGCCACTCCCATA
β-Actin-RT-F	TACGAGCTGCCTGACGGACA
β-Actin-RT-R	GGCTGTGATCTCCTTCTGCA
MCP-RT-F	GCACGCTTCTCTCACCTTCA
MCP-RT-R	AACGGCAACGGGAGCACTA
VP19-RT-F	TCCAAGGGAGAAACTGTAAG
VP19-RT-R	GGGGTAAGCGTGAAGACT
LITAF-RT-F	GATGCTGCCGTGTGAACTG
LITAF-RT-R	GCACATCCTTGGTGGTGTTG
Ec-IL-1β-F	AACCTCATCATCGCCACACA
Ec-IL-1β-R	AGTTGCCTCACAACCGAACAC
Ec-IL-6-F	GGTTGGTCCAAGGTGTGCTTA
Ec-IL-6-R	CTGGGATTGTCGAGGTCCTT
Ec-IL-8-F	GCCGTCAGTGAAGGGAGTCTAG
Ec-IL-8-R	ATCGCAGTGGGAGTTTGCA
Ec-TNFα-F	GTGTCCTGCTGTTTGCTTGGTA
Ec-TNFα-R	CAGTGTCCGACTTGATTAGTGCTT

## Data Availability

The data that support the findings of this study are available from the corresponding author upon reasonable request.

## Supplementary Materials

The following supporting information can be downloaded at: https://www.mdpi.com/article/10.3390/v14081680/s1. Figure S1: Analysis of Ec-CD proteins.
