# Supplementary figures and images for: Functional Analysis of the Cathepsin D Gene Response to SGIV Infection in the Orange-Spotted Grouper, Epinephelus coioides
Source: Viruses. 2022 Jul 29;14(8):1680. doi: 10.3390/v14081680 (PMC9413388; doi:10.3390/v14081680)

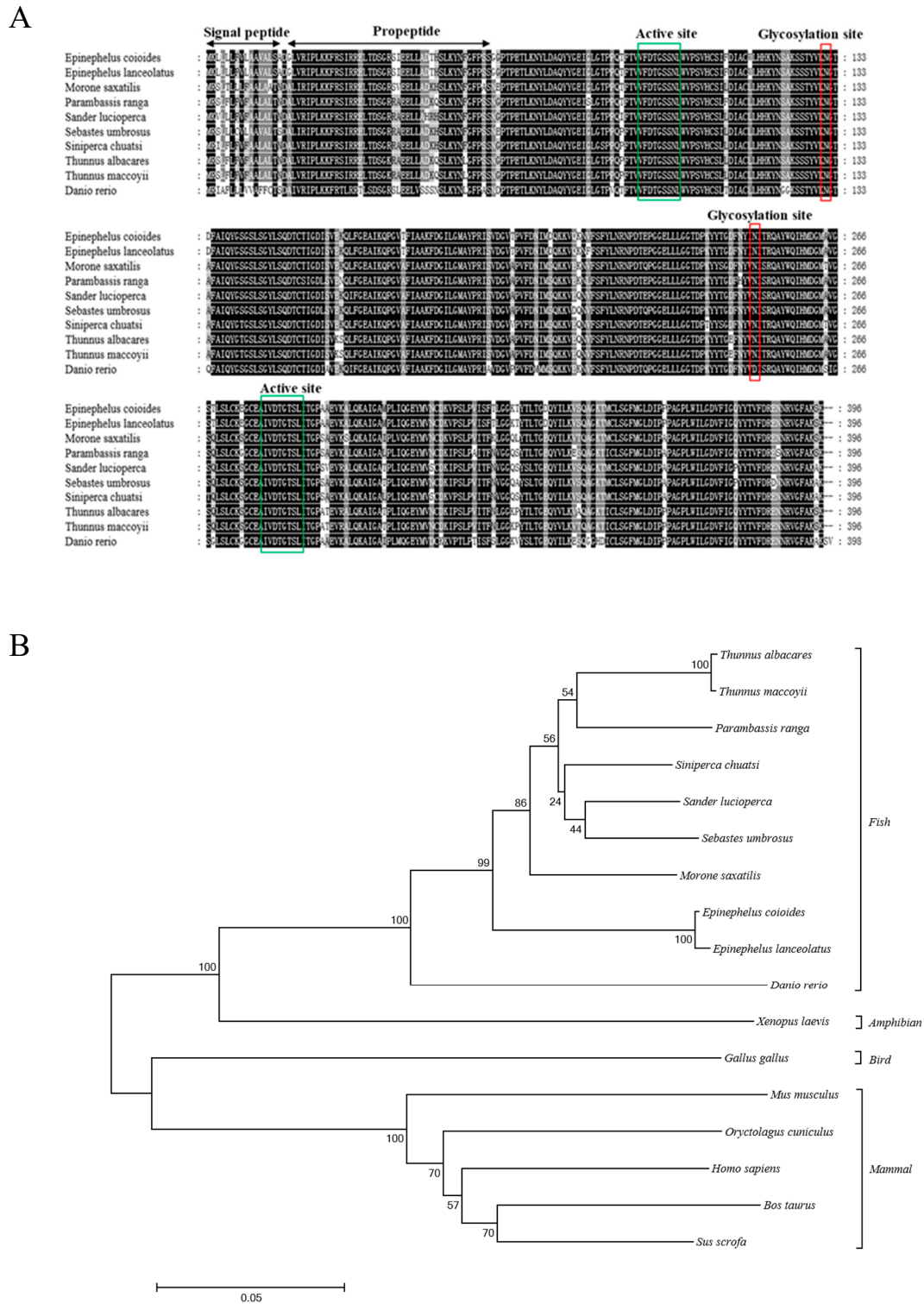

Supplement: Supplementary file 1 [file viruses-14-01680-s001.zip › viruses-1806542-supplementary.pdf]
